# The Nimodipine-Sparing Effect of Perioperative Dexmedetomidine Infusion During Aneurysmal Subarachnoid Hemorrhage: A Prospective, Randomized, Controlled Trial

**DOI:** 10.3389/fphar.2019.00858

**Published:** 2019-08-02

**Authors:** Chunguang Ren, Jian Gao, Guang jun Xu, Huiying Xu, Guoying Liu, Lei Liu, Liyong Zhang, Jun-Li Cao, Zongwang Zhang

**Affiliations:** ^1^Department of Anesthesiology, Liaocheng People’s Hospital, Liaocheng, China; ^2^Department of Neurosurgery, Liaocheng People’s Hospital, Liaocheng, China; ^3^Department of Anesthesiology, Xuzhou Medical University, Xuzhou, China

**Keywords:** dexmedetomidine, nimodipine, aneurysmal subarachnoid hemorrhage, aneurysm embolization, cerebral vasospasm

## Abstract

**Background:** Nimodipine can block the influx of calcium into the vascular smooth muscle cell and prevent secondary ischemia in patients with aneurysmal subarachnoid hemorrhage. However, the reduction of blood pressure after long-term intravenous administration of nimodipine has been associated with neurological deterioration. Yet, no effective solutions have been suggested to address this phenomenon. The use of neuroprotective drug combinations may reduce the risk of sudden blood pressure loss. This prospective, randomized, controlled trial was performed to evaluate the nimodipine-sparing effect of perioperative dexmedetomidine infusion during aneurysmal subarachnoid hemorrhage.

**Methods:** One hundred nine patients who underwent aneurysm embolization were divided into three groups: group C (*n* = 35, infused with 0.9% sodium chloride at the same rate as other two groups), group D1 (*n* = 38, dexmedetomidine infusion at 0.5 µg·kg^–1^ for 10 min, then adjusted to 0.2 µg·kg^–1^·h^–1^), and group D2 (*n* = 36, dexmedetomidine infusion at 0.5 µg·kg^–1^ for 10 min, then adjusted to 0.4 µg·kg^–1^·h^–1^). Patient-controlled analgesia was given for 48 h after surgery. The primary outcome measure was the total consumption of nimodipine during the first 48 h after surgery. The secondary outcome measures were recovery time at post-anesthesia care unit (PACU), postoperative pain intensity scores, dexmedetomidine and sufentanil consumption, hemodynamic, satisfaction of patients and neurosurgeon, neurologic examination (Glasgow Coma Scale, GCS), Bruggemann comfort scale, and adverse effects. Intraoperative hemodynamics were recorded at the following time-points: arrival at the operating room (T1); before intubation (T2); intubation (T3); 5 min (T4), 10 min (T5), and 15 min (T6) after intubation; suturing of femoral artery (T7); end of surgery (T8); extubation (T9); and 5 min (T10), 10 min (T11), and 15 min (T12) after arrival at the PACU. The level of sedation was recorded at 15 min, 30 min, 1 h, and 2 h after extubation. We also recorded the incidence of symptomatic cerebral vasospasm during 7 days after surgery, Glasgow Outcome Score (GOS) at 3 months, and incidence of cerebral infarction 30 days after surgery.

**Results:** The consumption of nimodipine during the first 48 h after surgery was significantly lower in group D2 (*P* < 0.05). Compared with group C, HR and MAP were significantly decreased from T2 to T12 in group D1 and D2 (*P* < 0.05). Patients in group D2 showed a significantly decreased MAP from T5 to T9 compared with group D1 (*P* < 0.05). The consumption of sevoflurane, remifentanil, dexmedetomidine, and nimodipine were all significantly reduced in groups D1 and D2 during surgery (*P* < 0.05). Compared with group C, MAP was significantly decreased in groups D1 and D2 during the first 48 h after surgery (*P* < 0.05). Compared with group C, consumption of sufentanil and dexmedetomidine at 1 h, pain intensity at 1 h, and 8 h after surgery were significantly decreased in groups D1 and D2 (*P* < 0.05). FAS was significantly higher in group D2 at 8 h, 16 h, and 24 h after surgery. LOS was significantly lower only in group D2 at 0.5 h after surgery (*P* < 0.05). Compared with group C, BCS was significantly higher group D2 at 4 h and 8 h after surgery (*P* < 0.05). There were no significant differences among the three groups in consumption of propofol, cisatracurium, fentanyl, and vasoactive drugs during operation, recovery time at PACU, satisfaction of patients and neurosurgeon, and number of applied urapidil and GCS during the first 48 h after surgery. The incidence of symptomatic cerebral vasospasm during 7 days after surgery, GOS of 3 months, and cerebral infarction after 30 days were also comparable among the three groups.

**Conclusions:** Dexmedetomidine (infusion at 0.5 µg·kg^–1^ for 10 min, then adjusted to 0.4 µg·kg^–1^·h^–1^ during the surgery) significantly reduced the total consumption of nimodipine during the first 48 h after surgery and promoted early rehabilitation of patients although the incidences of symptomatic cerebral vasospasm, GOS, and cerebral infarction were not reduced.

## Introduction

Aneurysmal subarachnoid hemorrhage (aSAH) is an acute cerebrovascular condition associated with high morbidity and mortality worldwide (Lindgren et al., 2018). It imposes a huge economic burden on public health systems since most patients need long-term care (Wong et al., 2016). According to WHO report, the mortality of patients with aSAH has reduced to 35%. However, complications following aSAH are still a major clinical course (Hughes et al., 2018). One survey shows that up to 45% of patients with aSAH may have an unfavorable outcome. One of the most important factors associated with poor outcomes is the delayed cerebral infarction (DCI), which usually occurs 3–14 days after aSAH because of prolonged cerebral vasospasm (CVS) (Boulouis et al., 2017). So far, there has been no effective treatment to prevent its occurrence as the mechanism is largely unknown (Shimamura and Ohkuma, 2014).

Previous studies have strongly suggested that nimodipine should be administered *via* enteral administration except in cases of impaired absorption or metabolism (Soppi et al., 2007; Soppi et al., 2012; Hänggi et al., 2015; Tallarico et al., 2018). However, a major argument for the parenteral route of administration has been proposed as it provides more reliable and stable plasma concentrations (Abboud et al., 2015; Scheller et al., 2016). According to the prevailing evidence, intravenous administration of nimodipine should be used with care as many outcomes such as hypotension may lead to neurological worsening (Ahmed et al., 2000).

Recent studies reported that dexmedetomidine may have a neuroprotective effect and is safe for neurosurgery such as craniotomy and carotid endarterectomy (CEA) (McCutcheon et al., 2006; Garavaglia et al., 2014; Alam et al., 2017; Duan et al., 2018). The general goal of interventional anesthesia in neurosurgery is to keep patients motionless to optimize the quality of images, to maintain hemodynamic stability to avoid the risk of aneurysm rerupture, and to protect the brain against ischemic injury (Prabhakar et al., 2017). Although several studies have shown that hypotension during anesthesia induction may pose harm to the patients and increase the risk of early and delayed neurological deficits, general anesthesia is still the first choice (Fan et al., 2017; Ciporen et al., 2018).

Following our search for English language articles published between 1980 and 2017 on MEDLINE, PubMed, Embase, and Cochrane Central Register of Controlled Trials using the terms of dexmedetomidine, nimodipine, aneurysmal subarachnoid hemorrhage, and cerebral vasospasm, we did not find any study on the nimodipine-sparing effect of perioperative dexmedetomidine infusion during aneurysmal subarachnoid hemorrhage. Therefore, we conducted this prospective, randomized, controlled trial on this topic.

## Material and Methods

### Patients

We obtained ethical approval from the Institutional Review Board of Liaocheng People’s Hospital to conduct this prospective, randomized, controlled trial. Written informed consent was obtained from patients or their guardian before participation in this trial. The trial was also registered at chictr.org (ChiCTR-IPR-16008494, 19/05/2016). All methods were performed in accordance with the relevant guidelines.

Patients admitted to our hospital within 72 h of initial subarachnoid hemorrhage from October 2016 to June 2018 were included in this trial if they met the following criteria: age 40–75 years, American Society of Anesthesiologists (ASA) grades I to III, diagnosed with aSAH using digital subtraction angiography (DSA), undergoing aneurysm embolization with general anesthesia, using patient-controlled analgesia (PCA) for at least 48 h after surgery. Patients who met the following criteria were excluded: reoperation within 48 h, taking drugs known to interact with nimodipine such as anticonvulsants within the last month before enrollment, serious systemic diseases of liver and kidney, psychiatric disorders, ischemic heart disease or second- or third-degree atrioventricular block, long-term abuse of alcohol, opioids, or sedative-hypnotic drugs, obesity (body mass index [BMI] >30 kg/m^2^), and operative time <1 h or >3 h.

### Randomization and Blinding

A computer-generated randomization table was used to allocate patients into three groups by an independent anesthetist before surgery: group D1 (*n* = 38, dexmedetomidine was infused at 0.5 μg·kg^–1^ for 10 min, then adjusted to 0.2 μg·kg^–1^·h^–1^), group D2 (*n* = 36, dexmedetomidine was infused at 0.5 μg·kg^–1^ for 10 min, then adjusted to 0.4 μg·kg^–1^·h^–1^), and group C (*n* = 35, 0.9% sodium chloride was infused at rate the same as the other two groups). A team of nurses who offered the Acute Pain Services were blinded to this trial, and they prepared the experimental drug (dexmedetomidine was diluted to 50 ml using 0.9% sodium chloride in groups D1 and D2) and the PCA pump (sufentanil (0.01 μg·kg^–1^·h^–1^) plus dexmedetomidine (0.1 μg·kg^–1^·h^–1^) programmed to deliver a bolus of 2 ml, with a background infusion of 2 ml·h^–1^, a lockout of 5 min, and 1-h limit of 16 ml). All anesthesiologists and neurosurgeons who participated in this trial were blinded.

### Anesthesia

All patients had not been premedicated. Peripheral venous was established in the left upper extremity before the start of anesthesia, then five-lead electrocardiogram, noninvasive blood pressure, oxygen saturation, temperature, and bispectral index (Aspect Medical System, Newton, MA, USA) were continuously monitored using an automated system (IntelliVue MP50, Philips, Amsterdam, the Netherlands). The anesthetic methods adopted are routinely used in our center (Su et al., 2016). Oxygen (100%) was administered *via* a facial mask at 4 L/min for 5 min. After the experimental drug (0.9% sodium chloride in group C or dexmedetomidine in groups D1 and D2) was infused for 10 min in three groups, fentanil (2–3 μg·kg^–1^), propofol (1.5–2 mg·kg^–1^), and cisatracurium (0.2 mg·kg^–1^) were administered intravenously, and tracheal intubation was performed 3 min later. Sevoflurane (1.5–2.1%), remifentanil (0.05–0.1 μg·kg^–1^·min^–1^), and nimodipine (5–20 μg·kg^–1^·h^–1^) were administered during surgery. Cisatracurium (0.05 mg·kg^–1^) was intermittently added to maintain muscle relaxation. The pressure of arterial carbon dioxide (PaCO_2_) was maintained at 35–40 mmHg during surgery. Sevoflurane and the experimental drugs were stopped when all of the coils were placed. Remifentanil was continued until the femoral artery was sutured. All patients received 5 mg of tropisetron and underwent routine reversal of neuromuscular blockade (atropine 4 μg·kg^–1^ plus neostigmine 15 μg·kg^–1^) at the end of surgery.

The concentration of sevoflurane was adjusted by 0.2% stepwise titration according to BIS. Remifentanil infusion was adjusted by 0.01-μg·kg^–1^·min^–1^ stepwise titration according to acceptable hemodynamic limits. Nimodipine was adjusted by 5-μg·kg^–1^·h^–1^ stepwise titration if premise of satisfactory depth of anesthesia and poor response to remifentanil. Besides, phenylephrine, ephedrine, atropine, and urapidil were used where necessary. Endovascular embolization was performed by the same neurosurgeon who had more than 15 years of residency experience.

### Postoperative Management

All patients underwent head computed tomography (CT) scan immediately after surgery to detect acute complications such as hemorrhage before they were transferred to the PACU. The same neurosurgeon performed neurological examination and trained the patients to operate the PCA pump. Patients were encouraged to push the bolus of PCA when VASm >3. For patients with poor response to PCA, 1 mg butorphanol was administered intravenously.

Nimodipine was infused at 5–20 μg·kg^–1^·h^–1^ to maintain hemodynamic parameters after surgery according to the standards of “Guidelines for the Management of Aneurysmal Subarachnoid Hemorrhage: A Guideline for Healthcare Professionals from the American Heart Association/American Stroke Association” (Lawton and Vates, 2017). The following interventions were performed if patients showed neurological symptoms in the postoperative period: positive concordance between clinical examination and transcranial Doppler ultrasound (TCD) results led to induction of hypertensive therapy, and discordance between clinical examination and TCD results led to further testing with CT or computed tomography angiography (CTA) (Jung et al., 2012). All patients received CTA as a routine examination at discharge.

### Data Collection

The primary outcome measure was the total consumption of nimodipine during the first 48 h after surgery. The secondary outcome measures were recovery time at PACU, the movement of postoperative pain intensity score (VASm), consumption of dexmedetomidine and sufentanil, hemodynamic satisfaction of patients and neurosurgeon, neurological examination (Glasgow coma scale, GCS), Bruggemann comfort scale (BCS: 0, persistent pain; 1, severe pain while deep breathing or coughing; 2, mild pain while deep breathing or coughing; 3, no pain while deep breathing; and 4, no pain while coughing) and adverse effects.

Intraoperative hemodynamic data (MAP and HR) were recorded at the following time-points: arrival at the operating room (T1); before intubation (T2); intubation (T3); 5 min (T4), 10 min (T5), and 15 min (T6) after intubation; suturing of femoral artery (T7); end of surgery (T8); extubation (T9); and 5 min (T10), 10 min (T11), 15 min (T12) after arrival at the PACU. Level of sedation (LOS: 0, fully awake; 1, drowsy/closed eyes; 2, asleep/easily aroused with light tactile stimulation or a simple verbal command; 3, asleep/arousable only by strong physical stimulation; and 4, unarousable) was recorded at 15 min and 30 min and 1 h and 2 h after extubation. We also recorded the incidence of symptomatic cerebral vasospasm during 7 days after surgery, Glasgow Outcome Score (GOS) at 3 months, and incidence of cerebral infarction 30 days after surgery.

### Statistical Analyses

According to our previous study, the sample size was calculated on the basis of an expected difference of 20% in the cumulative amount of nimodipine utilized 48 h after surgery. For a study power of 80% (α = 0.05, β = 0.2), the required sample size per group was calculated to be 32 (PASS 11.0, NCSS Statistical Software, Kaysville, Utah). Assuming a dropout rate of 10%, the final sample size was determined to be 35 patients for each group.

Statistical analyses were carried out using SPSS for Windows Version 21.0 (SPSS Inc. Chicago, IL, USA). The Kolmogorov–Smirnov test was used to assess the distribution of the variables. Homogeneity of variance was determined using Levene’s test. Normally distributed continuous variables were presented as mean ± SD. Inter-group comparisons were performed using repeated-measures analysis of variance. The Bonferroni’s correction was used for *post hoc* multiple comparisons. The nonparametric Kruskal–Wallis test was used for non-normally distributed continuous variables presented as an inter-quartile range. Categorical data were expressed as frequencies and percentages and analyzed using chi-squared tests or Fisher’s exact tests. *P* < 0.05 was considered statistically significant.

## Results

### Baseline Characteristics

**Figure 1:**
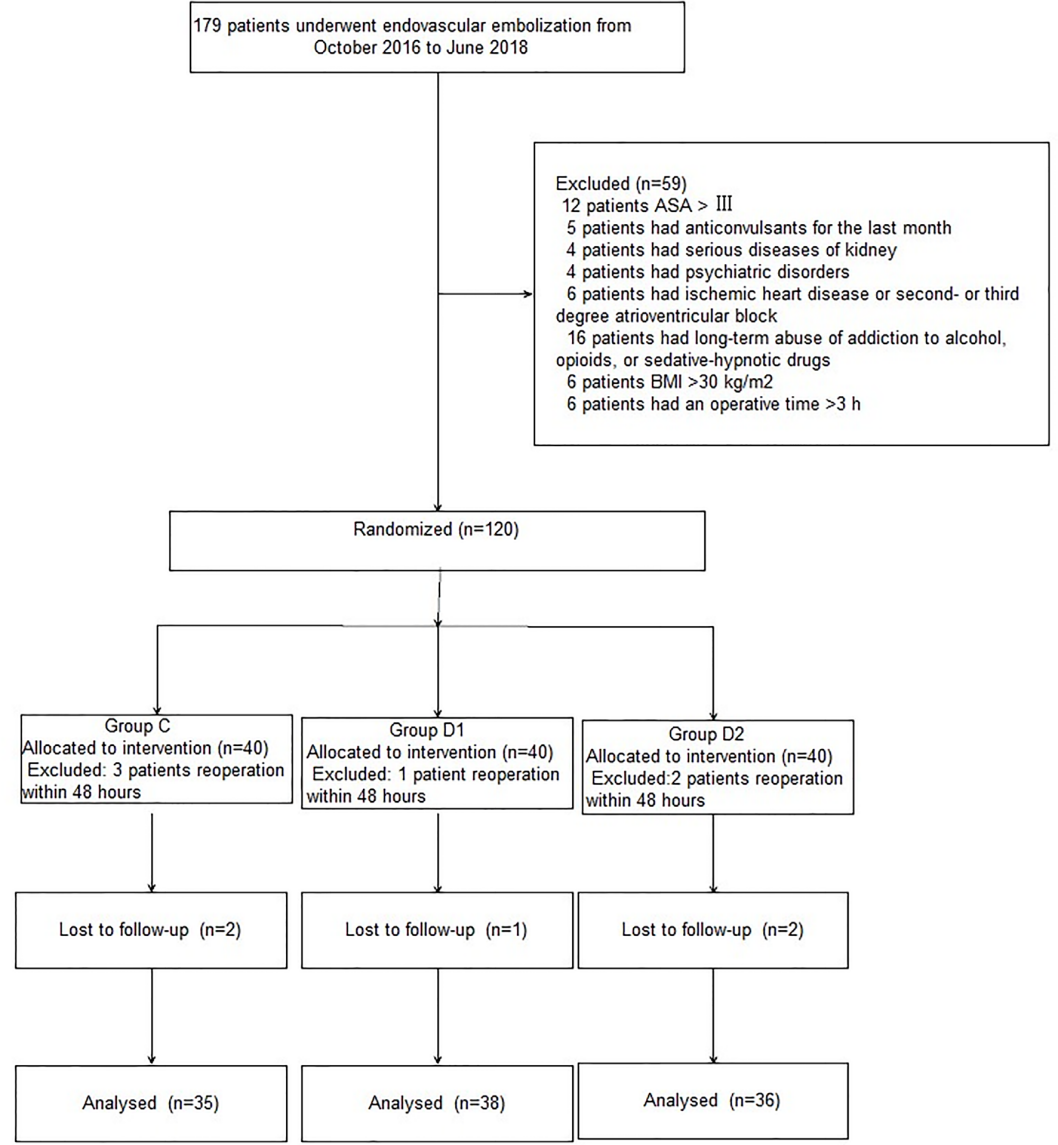
Patients enrollment flow diagram.

A CONSORT diagram was used during the enrollment of patients (**Figure 1**). One hundred seventy-nine patients who underwent aneurysm embolization from October 2016 to June 2018 were recruited. Fifty-nine patients were excluded: ASA of 12 patients >III; five patients had anticonvulsants in the last month; four patients had serious systemic diseases of kidney; four patients had psychiatric disorders; six patients had ischemic heart disease or second- or third-degree atrioventricular block; 16 patients had long-term abuse of alcohol, opioids, or sedative-hypnotic drugs; BMI of six patients >30 kg/m^2^; and six patients had an operative time >3 h. Finally, 120 patients were included in the primary analysis, among whom 11 were excluded (five patients from group C, two patients from group D1, and four patients from group D2). At last, 109 remaining patients were divided into three groups: 35 patients for group C, 38 patients for group D1, and 36 patients for group D2. Three groups were comparable in terms of age, BMI, ASA grade, sex, comorbidity, and GCS before surgery (*P* > 0.05) (**Table 1**).

**Table 1:** Clinical characteristics of patients in the three groups.

Variable	Group C (*n* = 35)	Group D1 (*n* = 38)	Group D2 (*n* = 36)	*P*-values
Age (years)	62.11 ± 5.87	61.79 ± 6.28	62.20 ± 7.54	.962
Body weight (kg)	67.29 ± 6.39	67.55 ± 5.73	66.81 ± 7.86	.890
BMI (kg·m^–2^)	23.91 ± 2.05	24.08 ± 2.25	23.44 ± 2.27	.441
ASA I/II/III (*n*)	5/21/9	6/26/6	7/22/7	.835
Sex (male/female)	17/18	19/19	21/15	.678
Comorbidity, *n* (%)				.995
Hypertension	17 (48.57%)	21 (55.26%)	14 (38.89%)	
Diabetes mellitus	6 (17.14%)	7 (18.42%)	6 (16.67%)	
Coronary heart disease	3 (8.57%)	4 (10.53%)	3 (8.33%)	
GCS before surgery	15.00 (14.00–15.00)	15.00 (14.00–15.00)	14.00 (14.00–15.00)	.685

Variables presented as mean ± SD, median (interquartile range) or number of patients n (%). BMI, body mass index; ASA, American Society of Anesthesiology; GCS, Glasgow Coma Scale.

### Intraoperative Variables

**Figure 2:**
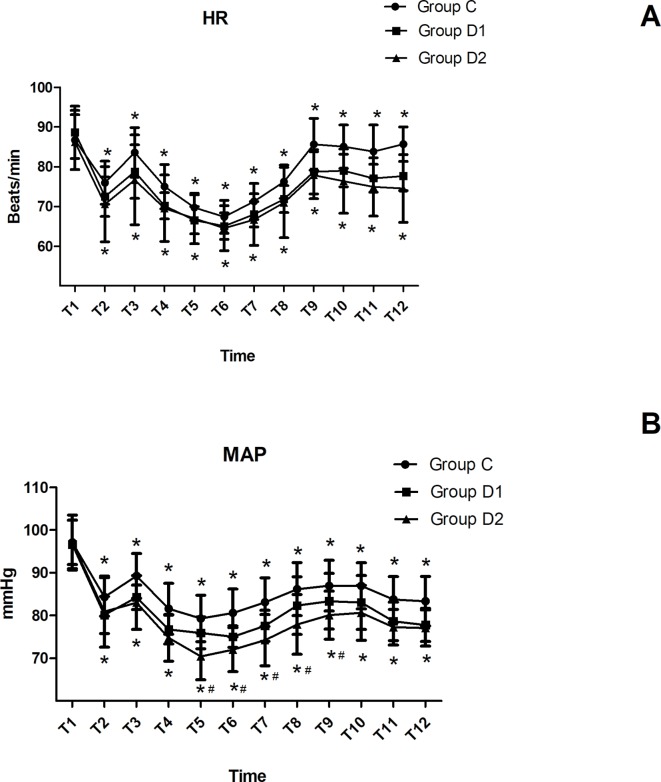
Hemodynamics changes during the surgical procedure and post-anesthesia care unit (PACU) stay. **(A)** Comparison of HR in the three groups at different time-points. **(B)** Comparison of MAP in the three groups at different time-points. *P < 0.05 *vs.* Group C, ^#^ *P* < 0.05 *vs.* Group D1.

HR and MAP were not significantly different among the three groups at arrival in the operating room (*P* > 0.05). Compared with group C, patients in groups D1 and D2 showed significantly decreased HR and MAP from T2 to T12 (*P* < 0.05). Patients in group D2 showed a significant decrease in MAP from T5 to T9 compared with group D1 (*P* < 0.05) (**Figure 2**).

Compared with group C, consumption of sevoflurane (1.70 [1.50–1.70] *vs.* 1.50 [1.50–1.70] *vs*. 1.50 [1.50–1.50] %, *P* = 0.007), remifentanil (628.24 ± 76.42 *vs.* 523.37 ± 73.66 *vs.* 453.37 ± 86.11 μg, *P* < 0.001), dexmedetomidine (0 *vs.* 61.74 [57.33–66.29] *vs.* 87.89 [81.37–95.72] μg, *P* < 0.001), and nimodipine (1.42 ± 0.17 *vs.* 1.13 ± 0.13 *vs.* 0.69 ± 0.20 mg, *P* < 0.001) were significantly reduced in groups D1 and D2—these differences were also statistically significant between groups D1 and D2 (**Table 2**). There were no significant differences among the three groups in the duration of surgery and anesthesia, consumption of propofol, cisatracurium, and fentanyl (*P* > 0.05) (**Table 2**). The number of patients using atropine, ephedrine, phenylephrine, and urapidil was comparable among the three groups during operation (**Table 3**).

**Table 2:** Comparison of intraoperative variables in the three groups.

Variable	Group C (*n* = 35)	Group D1 (*n* = 38)	Group D2 (*n* = 36)	*P*-values
Duration of surgery (min)	113.00 (105.00–121.00)	110.00 (102.00–119.25)	110.50 (102.50–122.75)	.770
Duration of anesthesia (min)	135.00 (125.00–146.00)	130.50 (126.00–140.50)	134.50 (127.25–147.50)	.694
Remifentanil dosage (µg)	628.24 ± 76.42	523.37 ± 73.66*	453.37 ± 86.11*^#^	.000
Dexmedetomidine dosage (µg)	—	61.74 (57.33–66.29)*	87.89 (81.37–95.72)*^#^	.000
Nimodipine dosage (mg)	1.42 ± 0.17	1.13 ± 0.13*	0.69 ± 0.20*^#^	.000
Propofol dosage (mg)	120.00 (110.00–130.00)	120.00 (110.00–140.00)	120.00 (110.00–147.50)	.697
Cisatracurium dosage (mg)	19.03 ± 3.71	19.42 ± 3.54	19.00 ± 3.93	.862
Fentanyl dosage (mg)	0.20 (0.20–0.25)	0.20 (0.20–025)	0.20 (0.20–0.20)	.233
Sevoflurane (%)	1.70 (1.50–1.70)	1.50 (1.50–1.70)*	1.50 (1.50–1.50)*^#^	.007

Variables presented as mean ± SD or median (interquartile range). *P <0.05 vs. Group C, ^#^P <0.05 vs. Group D1.

**Table 3:** Consumption of vasoactive drugs during operation.

Variable	Group C (*n* = 35)	Group D1 (*n* = 38)	Group D2 (*n* = 36)	*P*-values
Atropine	2 (5.71%)	3 (7.89%)	7 (19.44%)	.196
Ephedrine	3 (8.57%)	6 (15.79%)	6 (16.67%)	.607
Phenylephrine	5 (14.29%)	5 (13.16%)	3 (8.33%)	.758
Urapidil	4 (11.43%)	4 (10.53%)	2 (5.56%)	.708

Variables presented as number of patients n (%).

### Postoperative Variables

**Figure 3:**
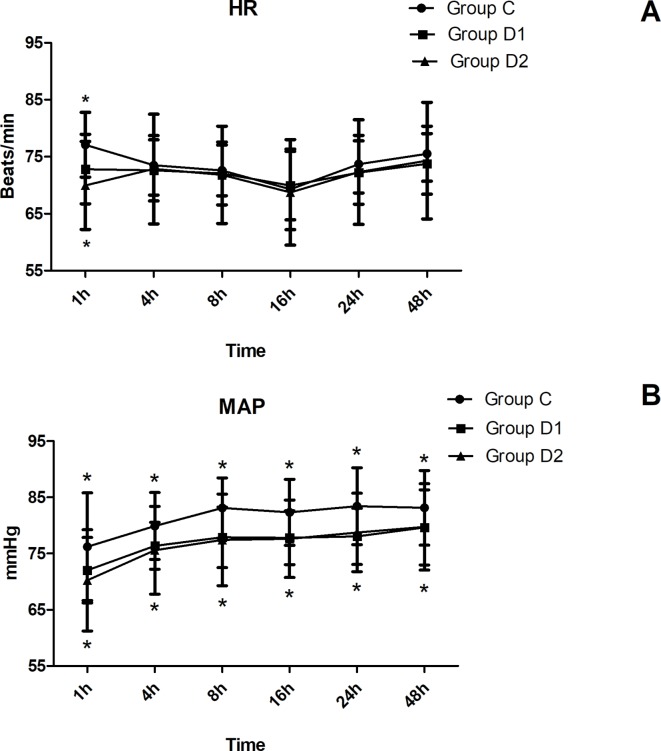
Hemodynamics changes during the first 48 h after surgery. **(A)** Comparison of HR in the three groups at different time-points. **(B)** Comparison of MAP in the three groups at different time-points. *P < 0.05 *vs.* Group C.

The consumption of nimodipine during the first 48 h after surgery was significantly lower in group D2 (63.36 [60.48–69.12] *vs.* 51.84 [48.38–55.30] *vs.* 31.92 [30.24–34.44] mg, *P* < 0.001) (**Table 4**). Compared with group C, HR at 1 h after surgery was significantly decreased in groups D1 and D2 (*P* < 0.05), while MAP was significantly decreased in groups D1 and D2 during the first 48 h after surgery (*P* < 0.05) (**Figure 3**). FAS was significantly higher in group D2 at 8 h (*P* = 0.021), 16 h (*P* = 0.007), and 24 h (*P* = 0.001) after surgery (**Table 5**).

**Table 4:** Consumption of postoperative variables in the three groups.

Variable	Group C (*n* = 35)	Group D1 (*n* = 38)	Group D2 (*n* = 36)	*P*-values
Recovery time at PACU (min)	16.00 (13.00–18.00)	15.00 (12.75–17.00)	15.00 (12.00–19.00)	.862
Nimodipine dosage (mg)	63.36 (60.48–69.12)	51.84 (48.38–55.30)*	31.92 (30.24–34.44)*^#^	.000
Patient satisfaction score	8.00 (8.00–9.00)	8.00 (8.00–9.00)	9.00 (8.00–9.00)	.055
Neurosurgeon satisfaction score	9.00 (8.00–9.00)	9.00 (8.00–9.00)	9.00 (8.00–9.00)	.996
Symptomatic cerebral vasospasm	2 (5.71%)	2 (5.26%)	2 (5.56%)	1.000
Number of applied urapidil, *n* (%)	9 (25.71%)	7 (18.42%)	4 (11.11%)	.288
GOS of 3 months (3/4/5)	4/13/18	2/13/23	3/15/18	.815
Cerebral infarction after 30 d, *n*(%)	8 (22.86%)	7 (18.42%)	5 (13.89%)	.642

Variables presented as median (interquartile range) or number of patients n (%). PACU, post-anesthesia care unit; GOS, Glasgow outcome scale. *P < 0.05 vs. Group C, ^#^P < 0.05 vs. Group D1.

**Table 5:** FAS during 48 h after surgery in the three groups.

	Variable	Group C (*n* = 35)	Group D1 (*n* = 38)	Group D2 (*n* = 36)	*P*-values
FAS: C/B/A (*n*)	1 h	35/0/0	38/0/0	36/0/0	1.000
	4 h	35/0/0	38/0/0	35/1/0	.651
	8 h	9/26/0	6/31/1	3/27/6*	.021
	16 h	0/26/9	2/27/9	0/16/20*^#^	.007
	24 h	0/13/22	0/2/36*	0/4/32*	.001
	48 h	0/0/35	0/0/38	0/0/36	1.000

Variables presented as number of patients n. FAS, functional activity score. *P <0.05 vs. Group C, ^#^P < 0.05 vs. Group D1.

**Figure 4:**
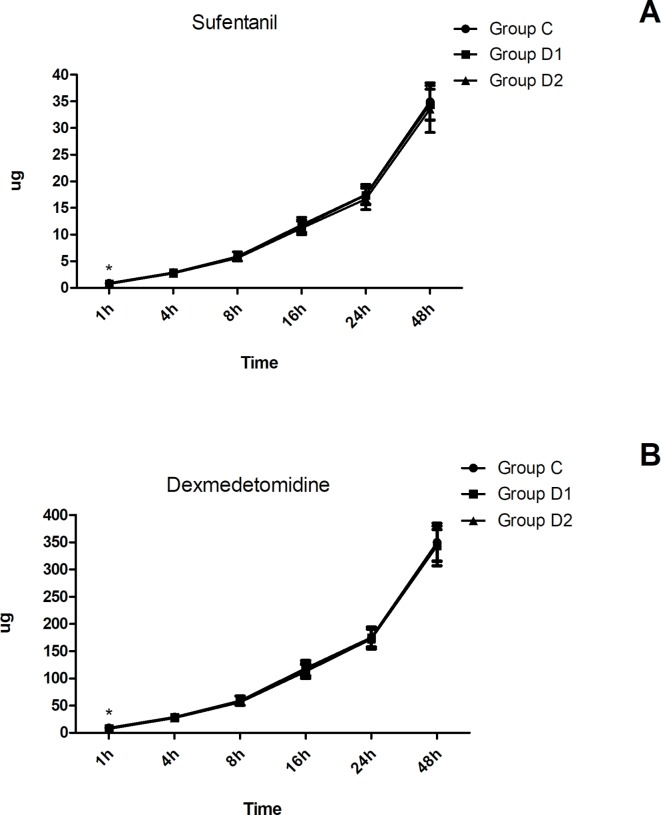
Postoperative consumption of sufentanil and dexmedetomidine in the three groups. **(A)** Postoperative consumption of sufentanil in the three groups at different time-points. **(B)** Postoperative consumption of dexmedetomidine in the three groups at different time-points. *P < 0.05 *vs.* group C.

**Figure 5:**
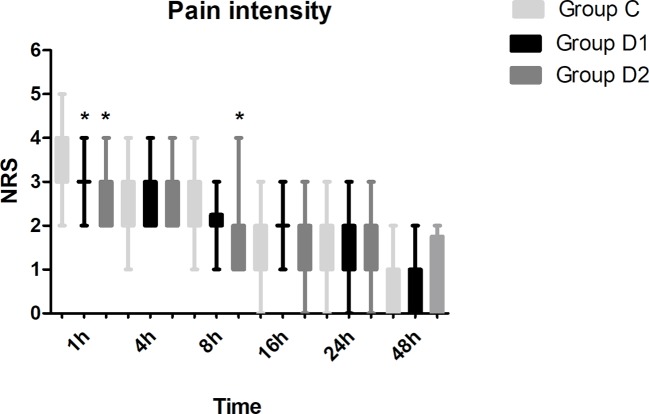
Postoperative pain intensity in the three groups expressed as scores on a numerical rating scale (NRS) out of 10. *P < 0.05 *vs.* group C.

**Figure 6:**
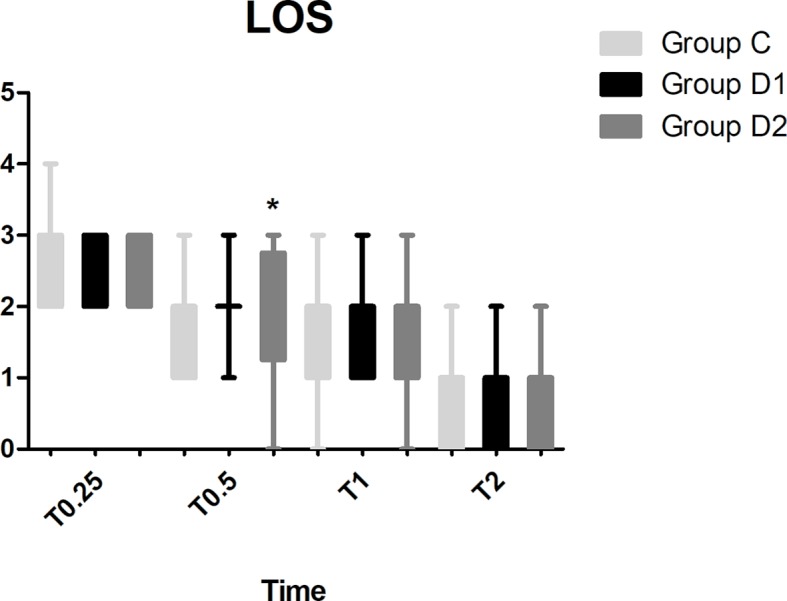
Comparison of patient sedation among the three groups using the level of sedation (LOS). LOS: 1, the subject is anxious, agitated, or restless; 2, the subject is cooperative, oriented, tranquil, and responds to commands; 3, the subject is asleep but has a brisk response to light glabellar tap or a loud auditory stimulus; 4, the subject is asleep, has a sluggish response to a light glabellar tap or loud auditory stimulus; 5, the subject is asleep and unresponsive. *P < 0.05 *vs.* group C.

**Figure 7:**
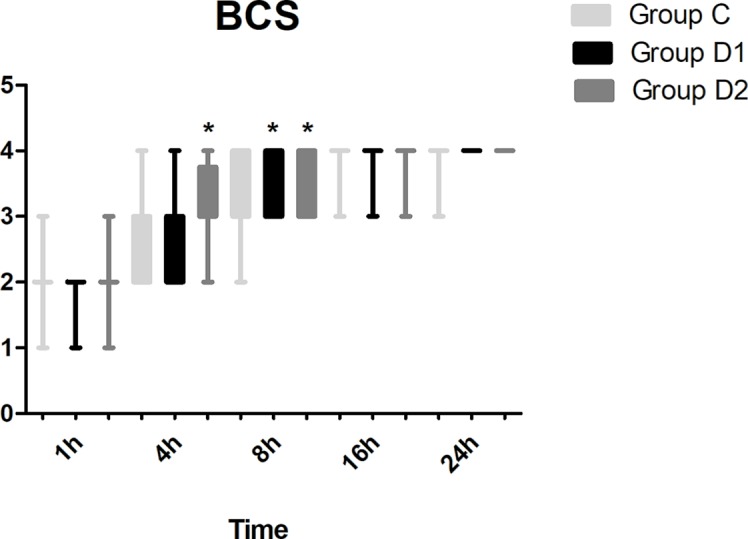
Comparison of Bruggemann Comfort Scale (BCS) scores among the three groups. BCS: 0, persistent pain; 1, severe pain while deep breathing or coughing; 2, mild pain while deep breathing or coughing; 3, painless while deep breathing; 4, painless while coughing. *P < 0.05 *vs.* group C.

**Figure 8:**
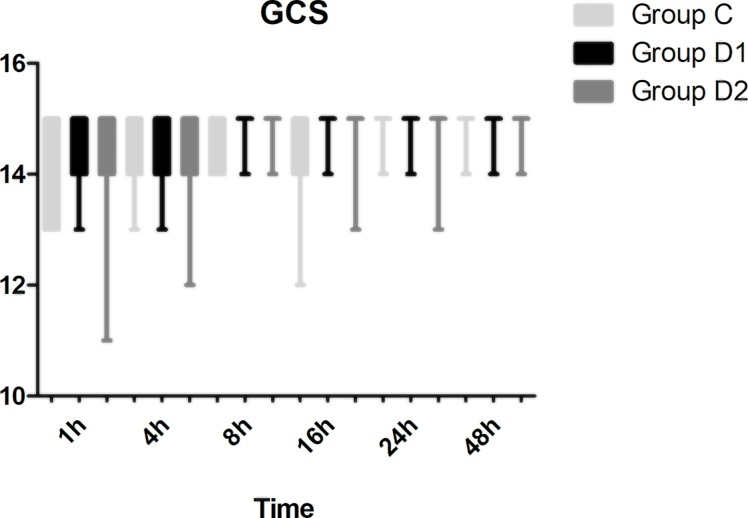
Comparison of the Glasgow Coma Scale (GCS) scores among the three groups.

Compared with group C, consumption of sufentanil and dexmedetomidine at 1 h after surgery was significantly decreased in groups D1 and D2 (*P* < 0.05) (**Figure 4**). Compared with group C, the pain intensity was only significantly lower at 1 h and 8 h after surgery (*P* < 0.05) (**Figure 5**). LOS was significantly lower only in group D2 at 0.5 h after surgery (*P* < 0.05) (**Figure 6**). Compared with group C, BCS was significantly higher in group D2 at 4 h and 8 h after surgery (*P* < 0.05) (**Figure 7**). There were no significant differences among the three groups in recovery time at PACU, satisfaction of patients and neurosurgeon, number of patients given urapidil, and GCS during first 48 h after surgery (*P* > 0.05) (**Table 4**, **Figure 8**). The incidence of symptomatic cerebral vasospasm during 7 days after surgery, GOS at 3 months, and cerebral infarction after 30 days were comparable among the three groups (**Table 4**). Additionally, there were no significant differences among the three groups in adverse effects such as nausea, dizziness, thirst, hypotension, and hypertension (**Table 6**).

**Table 6:** Postoperative adverse effects of patients in the three groups.

Variable	Group C (*n* = 35)	Group D1 (*n* = 38)	Group D2 (*n* = 36)	*P*-values
Nausea	4 (11.43%)	4 (10.53%)	3 (8.33%)	.926
Dizzy	5 (14.29%)	2 (5.26%)	3 (8.33%)	.376
Thirst	—	—	3 (8.33%)	.065
Hypertension	7 (20.00%)	5 (13.16%)	4 (11.11%)	.556
Hypotension	3 (8.57%)	4 (10.53%)	1 (2.78%)	.480

Variables presented as number of patients n (%).

## Discussion

We report, for the first time, that high dose of dexmedetomidine (infusion at 0.5 μg·kg^–1^ for 10 min, then adjusted to 0.4 μg·kg^–1^·h^–1^ during the surgery) significantly reduced the total consumption of nimodipine during the first 48 h after surgery without any related complications. We also found that the requirements of sevoflurane, remifentanil, and nimodipine in group D2 were significantly decreased during the operation. Although more patients in group C needed vasoactive drugs during the first 48 h after surgery, there was no significant difference among the three groups. Compared with group C, FAS at 8–24 h, BCS at 4–8 h, pain intensity at 1 h, and 8 h after surgery were significantly improved in group D2. LOS in group D2 was significantly lower at 0.5 h after surgery, which may be attributed to the lower consumption of sufentanil and dexmedetomidine. We found no significant differences among the three groups in recovery time at PACU, the satisfaction of patients and surgeon, GCS, the incidence of symptomatic cerebral vasospasm during 7 days after surgery, GOS at 3 months, and cerebral infarction after 30 days.

Statistically, aSAH accounts for about 5% of all strokes patients. The suspected high-risk factors include hypertension, smoking, alcohol abuse, the use of sympathomimetic drugs, being female, unruptured cerebral aneurysm, a history of previous aSAH, and familial aneurysms (Tallarico et al., 2018). Mortality due to this condition was remarkably reduced from 1970 to 1990 due to the development of new diagnostics and treatments (Kellner et al., 2012). Although treatment of high blood pressure with antihypertensive medication is recommended by AHA and ASA at the level of evidence A to prevent serious complications, only oral nimodipine is recommended for all patients with aSAH to improve neurological outcomes (Connolly et al., 2012). In our center, aSAH patients are routinely admitted to the neurosurgery intensive care unit (NICU) and managed by a multidisciplinary team. They are regularly treated with intravenous infusion of nimodipine at 1–2 mg·h^–1^ or enteral administration of 60 mg orally at 4 h intervals. If the systolic blood pressure <110 mm Hg, the infusion rate of nimodipine is reduced to 0.5–1 mg·h^–1^ according to previous studies. The consumption of nimodipine during the first 48 h after surgery was lower than previous studies, possibly due to the use of different drug combinations (Zarauza et al., 2000; Casey et al., 2006).

33.7% of patients with SAH have an unfavorable outcome because of cerebral vasospasm caused by an increase of calcium level in the vascular smooth muscle cells. However, the percentage of patients with unfavorable outcome has been underestimated in many studies due to the exclusion of patients who die before treatment (Konczalla et al., 2016). Here, we only recorded the incidence of symptomatic cerebral vasospasm 7 days after surgery since the highest risk period for cerebral vasospasm usually occurs 3–14 days after aSAH and the duration of discharge after surgery was within 8 days for most patients in our center (Oertel et al., 2005). The commonly used methods to monitor cerebral vasospasm include clinical, physiological, and radiographic. Clinical examination is generally thought to be suitable for good-grade patients. Physiological monitoring includes transcranial TCD, electroencephalography (EEG), brain tissue oxygen monitoring, cerebral microdialysis, thermal diffusion cerebral blood flow (TD-CBF), and near-infrared spectroscopy. Additionally, DSA, CTA/CTP, CTA-MMBE (CTA combined with matched mask bone elimination), and dual-energy CTA are the common radiographic examinations (Bacigaluppi et al., 2015; Rao and Muthuchellappan, 2016). Sanelli et al. suggested that CTA and CTP are the preferred imaging strategy than TCD in aSAH as they improve the clinical outcomes and lower health care costs. However, DSA is still the gold standard for diagnosing cerebral vasospasm although it carries some risk of vascular complications such as thromboembolism and dissection (Sanelli et al., 2014). Similar to a previous study, we first performed clinical examination before further monitoring in this trial to reduce medical costs (Bricout et al., 2015). As a result, we only recruited patients of ASA I to III. However, previous studies recommend that radiographic and/or physiological monitoring should be routinely performed for SAH patients during ‘‘at risk’’ period even in the absence of clinical evidence of DCI. This may explain the lower incidence of symptomatic cerebral vasospasm observed 7 days after surgery in this study (Francoeur and Mayer, 2016). In our trial, we also evaluated the occurrence of cerebral infarction 30 days after surgery with TCD or CT/CTA and the incidence was similar to that of a previous study (Chhor et al., 2011).

Previous studies have proposed the use of calcium channels blockers to prevent secondary ischemia as these drugs can suppress the influx of calcium into the vascular smooth muscle cell, thereby decreasing the rate of vasospasm (Tjahjadi et al., 2013). Systematic reviews found that endovascular treatments such as intra-arterial injection of pharmacological agents e.g., nimodipine or papaverine and balloon angioplasty, may improve the outcome of patients with severe-refractory vasospasm (Zwienenberg-Lee et al., 2008; Kieninger et al., 2018). Contrary to this conclusion, a previous study showed that nimodipine administration, specifically by intra-arterial bolus application, is associated with a reduction in MAP and CPP, which will require high doses of vasopressors to maintain cerebral perfusion. In addition, phlebitis at the injection site and pulmonary edema in addition to azotemia may occur as complications (Adami et al., 2019). Unfortunately, we did not record these complications in our trial for the lack of human and financial resources.

Niaz et al. found that the reduction of diastolic pressure (>20% reduction) was associated with neurological worsening after intravenous administration of 2 mg·h^–1^ nimodipine within 24 h after acute stroke, partly because of the higher incidence of cardiac ischemia. As a result, intravenous high-dose nimodipine (2 mg·h^–1^) seemed to be outweighed by the hemodynamic effect. Hence, the author proposed that administration of a plasma expanding drug could be an appropriate strategy to reduce the risk of sudden initial BP reactions (Niaz and Nayyar, 2003). We also monitored the hemodynamic changes 48 h after surgery as Heiss found that the BP change in the first 2 days after surgery was sufficient for analysis of neurological and functional outcome (Heiss, 2017). Only one patient in group D2 had hypotension in this trial, and the differences among the three groups did not reach statistical significance. The incidence of hypotension in group D2 was lower than that of the previous study, which may be attributed to the lower consumption of nimodipine and the synergistic effects of nimodipine and dexmedetomidine (Konczalla et al., 2016). Dexmedetomidine, a new highly selective agonist of α2 adrenergic receptor, is widely used in neurosurgery such as craniotomy, CEA for sedation, analgesia, and low risk of respiratory depression. A recent study reported that dexmedetomidine produced neuroprotective effects although the specific mechanism is still not known (Perez-Zoghbi et al., 2017). However, we did not observe this phenomenon possibly due to the lower dose of dexmedetomidine used in this trial.

Since 1991, an increasing number of patients choose intravascular embolization to treat aSAH. Compared with surgical clipping, both short- and long-term morbidity and mortality rates are reduced in patients treated with endovascular coiling (Tamargo et al., 2001). We recruited patients who were admitted to our hospital within 72 h of the initial hemorrhage event since decreased in-hospital mortality may be associated with early aneurysm treatment. Intraprocedural aneurysm rupture during endovascular coiling may lead to rapid increase in intracranial pressure. Hyperventilation and osmotic diuresis are the routinely used methods to control intracranial hypertension. In this trial, MAP was maintained at ±20% of baseline to avoid ischemia. In patients with intact autoregulation, administration of norepinephrine and epinephrine was found to be more suitable to maintain cerebral perfusion pressure compared with dopamine (Sookplung et al., 2011). Raabe et al. reported that moderate hypertension induced by phenylephrine may improve brain tissue oxygen pressure values (Raabe et al., 2005). Hence, we adopted phenylephrine and ephedrine to address hypotension in this trial.

Additionally, McNeill showed that the 6-month mortality of SAH is inversely related to the treatment institution. High-volume hospitals (>60 cases per year) improve the outcomes of patients by provision of neurocritical care units (McNeill, 2007). In our hospital, more than 400 patients undergo endovascular treatment every year and many specialized multidisciplinary teams have been established since 2012, which includes specialized NICU nurses, vascular neurosurgeons, interventional neuroradiologists, neuroanesthesiologist, and neurointensivists. Hence, the percentage of favorable outcomes in our hospital is about 40%, which is closer to the global levels. Hypernatremia, hyponatremia, and elevated blood glucose concentration are frequent complications in the acute phase after aSAH (Priebe, 2007). In our trial, the electrolyte level at 48 h after surgery was within normal limits because we recruited good-grade patients.

There are several limitations in this trial. First, the number of patients included in this trial is small and hence these results need to be confirmed by larger multicenter randomized controlled trials. Second, we did not measure the serum concentration of dexmedetomidine in this trial as a result of high hospital costs. Third, this trial only studied one dosage of dexmedetomidine-sufentanil after neurosurgery; different dosages should be further investigated. Finally, further studies on specific clinical monitoring for SAH patients are needed, especially for poor-grade patients.

In summary, we report for the first time, that high dose of dexmedetomidine (infusion at 0.5 μg·kg^–1^ for 10 min, then adjusted to 0.4 μg·kg^–1^·h^–1^ during surgery) significantly reduced the total consumption of nimodipine during the first 48 h after surgery and promoted early rehabilitation of patients although the incidence of symptomatic cerebral vasospasm, GOS, and cerebral infarction were not reduced. However, more multi-center prospective studies are required to determine the optimal dosage of dexmedetomidine during the perioperative period of neurosurgery.

## Data Availability

All datasets generated for this study are included in the manuscript and the supplementary files.

## Ethics Statement

All procedures performed in studies involving human participants were in accordance with the ethical standards of the institutional and/or national research committee and with the 1964 Helsinki declaration and its later amendments or comparable ethical standards.

## Author Contributions

CR, JG, GX, LZ, JC and ZZ conceived and designed the trial; JG and HX analyzed the data; CR, GL and LL collected the data, CR, JG, GX, HX, ZZ wrote this paper. JC and ZZ contributed equally to and should be considered co-corresponding author.

## Funding

This work was supported by the Natural Science Foundation of Shandong Province (ZR2016HB28).

## Conflict of Interest Statement

The authors declare that the research was conducted in the absence of any commercial or financial relationships that could be construed as a potential conflict of interest.
